# Development and psychometric evaluation of an instrument to measure knowledge, skills, and attitudes towards quality improvement in health professions education: The Beliefs, Attitudes, Skills, and Confidence in Quality Improvement (BASiC-QI) Scale

**DOI:** 10.1007/s40037-019-0511-8

**Published:** 2019-05-16

**Authors:** Allison Brown, Aditya Nidumolu, Meghan McConnell, Kent Hecker, Lawrence Grierson

**Affiliations:** 1 Michael G. DeGroote School of Medicine, McMaster University, Hamilton, ON Canada; 2 Department of Health Research Methods, Evidence, and Impact, McMaster University, Hamilton, ON Canada; 3 Department of Innovation in Medical Education, University of Ottawa, Ottawa, ON Canada; 4 Department of Community Health Sciences, University of Calgary, Calgary, AB Canada; 5 Department of Family Medicine, McMaster University, Hamilton, ON Canada; 6 McMaster Education Research, Innovation & Theory, McMaster University, Hamilton, ON Canada

**Keywords:** Quality improvement, Undergraduate medical education, Measurement

## Abstract

**Introduction:**

Health professionals are increasingly expected to foster and lead initiatives to improve the quality and safety of healthcare. Consequently, health professions education has begun to integrate formal quality improvement (QI) training into their curricula. Few instruments exist in the literature that adequately and reliably assess QI-related competencies in learners without the use of multiple, trained raters in the context of healthcare. This paper describes the development and psychometric evaluation of the Beliefs, Attitudes, Skills, and Confidence in Quality Improvement (BASiC-QI) instrument, a 30-item self-assessment tool designed to assess knowledge, skills, and attitudes towards QI.

**Methods:**

Sixty first-year medical student participants completed the BASiC-QI and the Quality Improvement Knowledge Application Tool (QIKAT-R) prior to and immediately following a QI program that challenged learners to engage QI concepts in the context of their own medical education. Measurement properties of the BASiC-QI tool were explored through an exploratory factor analysis and generalizability study. Convergent validity was examined through correlations between BASiC-QI and QIKAT-R scores.

**Results:**

Psychometric evaluation of BASiC-QI indicated reliability and validity evidence based on internal structure. Analyses also revealed that BASiC-QI scores were positively correlated with the scores from the QIKAT-R, which stands an indicator of convergent validity.

**Conclusion:**

BASiC-QI is a multidimensional self-assessment tool that may be used to assess beliefs, attitudes, skills, and confidence towards QI. In comparison with existing instruments, BASiC-QI does not require multiple raters or scoring rubrics, serving as an efficient, reliable assessment instrument for educators to examine the impact of QI curricula on learners.

**Electronic supplementary material:**

The online version of this article (10.1007/s40037-019-0511-8) contains supplementary material, which is available to authorized users.

## What this paper adds

Quality improvement (QI) training is important in health professions education, yet the evaluation of QI learning may be resource intensive, requiring a considerable amount of time and raters. The Beliefs, Attitudes, Skills, and Confidence in Quality Improvement (BASiC-QI) is a 30-item instrument which can assess knowledge, skills, and attitudes towards QI. BASiC-QI scores were positively correlated with scores from the current gold standard instrument for QI evaluations, the QIKAT-R. In comparison with existing tools, BASiC-QI is a less resource-intensive instrument that may be used to evaluate QI curricula.

## Introduction

Quality improvement (QI) training is an important component of health professions education that ensures that future health professionals can contribute to the advancement of healthcare, patient safety, and the overall performance of health systems [1–3]. QI and patient safety are recognized as cross-cutting competencies by the Canadian Medical Education Directives for Specialists (CanMEDS) Competency Framework, and a Common Program Requirement of the Accreditation Council for Graduate Medical Education (ACGME) in the United States [4, 5]. In this regard, there are numerous QI training programs that aim to develop QI competence in medical learners. Most of these programs engage learners as they move into the clinical practice components of their training (i.e., clerkship, residency) [2, 3]. We recognize there are potential benefits to initiating QI training for medical learners prior to clinical experiences based on our experiences with the Program for Improvement in Medical Education (PRIME) at McMaster University (Hamilton, Canada), which introduces the fundamentals of QI in the context of education [6, 7]. During this elective program, first-year medical students participate in a 3-hour extracurricular workshop that introduces QI in healthcare, but challenges participants to apply these concepts to medical education. Students then work in small groups to identify an area for improvement in the medical school curriculum and submit a project charter that outlines how they would use the Model for Improvement to design, test, and implement a change [6, 7]. The central idea for a program such as PRIME is that early exposure to the key concepts of QI positions learners to get the most out of the subsequent QI clinical experiences. Evaluations of PRIME have demonstrated improvements in medical students’ knowledge, skills, and attitudes towards QI following completion of the program [6, 7].

The importance of QI competence to healthcare delivery and the development of appropriate training programs is concomitant with the recognition that there is a need to assess medical learners in a way that ensures they have acquired the necessary knowledge, skills, and attitudes that will facilitate their involvement in QI initiatives upon completion of their training. In this regard, there are a number of tools that have been shown to exhibit reliability and validity evidence, such as the Quality Improvement Knowledge Application Tool (QIKAT-R) [8], the Assessment of Quality Improvement Knowledge and Skills (AQIKS) instrument [9], and the Mayo Evaluation of Reflection on Improvement Tool (MERIT) [10]. In each case, the research has shown these tools to have some utility; however, they are resource intensive. The use of multiple faculty raters to score these established instruments imposes resource requirements that may not be available within programs that wish to evaluate QI programming. Faculty raters must possess baseline knowledge of QI competencies in order to assess the quality of each participant’s response using the standardized rubric or scoring framework. In addition to requiring a substantial amount of time from participants to complete, these instruments similarly require a considerable amount of time from faculty raters to assess responses—especially if administered on several occasions across a training program.

Furthermore, the contextually bound nature of these tools is particularly challenging when one considers the assessment of QI knowledge, skills, and attitudes that are developed via training programs that are situated outside of the clinical context (e.g., PRIME). In situations such as these, these tools lack utility because their development is predicated on application testing and validity evidence derived from clinical settings [6, 7, 9]. Given the importance of QI training in health professions education, new assessment tools are needed that can reliably measure knowledge, skills, and attitudes following educational interventions. The purpose of our research was to develop an instrument which could be used to evaluate QI curricula at any stage of training—in any context—and to establish its measurement properties in order to highlight its potential for use in future evaluations of educational programming. The product of the present study is the Beliefs, Attitudes, Skills, and Confidence in Quality Improvement Scale (BASiC-QI): a 30-item tool designed to assess knowledge, skills, and attitudes towards QI (Supplement A of the Electronic Supplementary Material).

## Methodology

### Instrument development

The initial determination of the constructs that would underpin the BASiC-QI was facilitated through comprehensive review of the literature regarding existing QI curricula in health professions education at both the undergraduate and postgraduate levels, QI competencies, educational competencies assessment in medical learners, and QI assessment tools [3, 4, 8–12]. This resulted in an instrument that aimed to assess the ability of learners to understand and apply basic concepts of established QI methodologies, such as the Model for Improvement [13]. This aligns with the nature of the assessment of learners in most QI training programs, albeit always specifically with respect to the context of healthcare. For example, the QIKAT-R is designed to assess a participant’s ability to follow the Model for Improvement, which includes developing an aim statement, defining measures, and identifying a change concept or intervention; however, each QIKAT-R scenario describes a problem in the health system, to which individuals early in their training may have limited exposure.

With the initial items assembled, a focus group was held in November 2015 that queried 7 upper-year medical students from McMaster University who had previously participated in QI curricula about their perceptions of the key learning points derived from their training. These perceptions were compared against the items identified from the literature and used to refine the items included in the developed scale. This informed the inclusion of the Institute of Medicine’s ‘six dimensions of quality,’ ‘identifying a quality gap’ and ‘how to approach QI projects’ [14]. This was followed by key informant interviews with two leaders in QI education at McMaster University. These interviews focused on the anticipated impact of QI training and resulted in further refinement of the scale so as to include consideration for participant values and interest in QI. The idea was that by including attitudes and beliefs towards QI, the scale would be able to assess the likelihood that learners would engage in QI activities later in their training and career.

The result was an instrument with 37 items that were categorized under three subscales, each reflecting a construct for assessment. The first construct—*Beliefs and Attitudes*—included both feelings and perceptions about QI. The second construct—*Knowledge of QI*—included knowledge of broad concepts as well as hallmarks of all QI training (i.e., systems thinking, the Model for Improvement). The third construct—*QI Skills*—includes participants’ ability to execute or apply QI, including how to approach a problem and apply their own skills to generate change and lead to a sustained improvement.

### Content validation

A content coverage matrix was used to ensure that each construct had a sufficient number of items [15]. Items that were considered redundant to others were eliminated or combined. Items were worded such that they could be utilized by all health professions. Within the ‘*Knowledge of QI*’ and ‘*QI Skills*’ subscales, questions are listed in the sequential order by which one would design a QI project—from identifying a deficit in quality, understanding the problem and root causes, designing a change or intervention, then flowing through the Model for Improvement to test, implement, spread and scale a change, and measure impact using a family of measures [13]. Although several of the items included technical jargon (e.g., Plan-Do-Study-Act (PDSA) cycles), this language was considered germane to QI practice and deemed necessary to include within BASiC-QI in order to properly assess participants’ knowledge of the fundamental concepts. Further, several of the items could be simply assessed by asking participants to write in a response (i.e., list the six dimensions of quality, write an aim statement, etc.); however, the goal was to develop an instrument that could efficiently assess the basic level of knowledge, skills, and attitudes towards QI recognizing that some programs may be limited in faculty capacity, resources, or content expertise typically required to score assessment tools.

Content relevance of each item was conducted by surveying five QI experts who had experience in teaching, participating, or leading QI using a 5-point scale, which ranged from ‘*not relevant whatsoever*’ to ‘*extremely relevant*’. Items that did not have at least 4 of the 5 respondents ranking as ‘*extremely relevant*’ or ‘*somewhat relevant*’ were removed from the scale. This technique reduced the number of items by 7, resulting in a final scale with 30 items. All constructs in the content coverage matrix were adequately covered by 3 or more items. Next, a cognitive interviewing technique, which asked two individuals (one with prior QI experience, one with no prior QI exposure) to rephrase each item in the way they understood it, was used to improve the comprehension and interpretability of each item. This resulted in slight modifications to the phrasing and wording of the items. The final 30-item instrument included 9 items within the *Beliefs and Attitudes *subscale, 9 items within the *Knowledge of QI* subscale, and 12 items within the *QI Skills* subscale.

### Scaling responses

A combination of 7‑point Likert and adjectival scaling options were selected as response options, requiring subjects to select the single most appropriate response option for each item. Likert response options were selected for the *Beliefs and Attitudes* and *Knowledge of QI* subscales. For the *Beliefs and Attitudes* subscale, it was possible that participants may respond with disagreement to the statements. For the *Knowledge of QI *subscale, the intent was to measure the degree to which each participant believes they are knowledgeable in areas of QI. Once again, it was anticipated that some may respond with disagreement to the statements, especially prior to any QI training. Adjectival scaling responses seemed especially appropriate for the *QI Skills* subscale and the assessment of participants’ confidence in their own skills relating to QI, as this is a continuum of confidence for assessment rather than a bipolar response option. For both the *Knowledge of *QI and *QI Skills* subscales, the Dreyfus model of skill acquisition was used to frame these constructs as stages through which learners should progress [16, 17]. Seven items were selected to allow for a wide range of response options, ideally allowing for variability to be captured between participants and time periods to give a better understanding of the impact if administered in a repeated measures design. Both *Beliefs and Attitudes *and *Knowledge of QI* subscales can be scored out of 63, while the *QI Skills* is scored out of 84. The total score possible on BASiC-QI when the subscales are combined is 210.

### Instrument testing

To test the measurement properties of BASiC-QI, the instrument was administered to 60 first-year medical students at McMaster University who completed the PRIME [6, 7]. Participants completed two instruments prior to and following PRIME: BASiC-QI and the QIKAT-R. During both administrations, participants completed the QIKAT-R prior to completing the BASiC-QI instrument. This meant that participants had to apply QI concepts through the QIKAT-R, and then reflect on their current knowledge, skills, and attitudes while completing the BASiC-QI instrument. Each participant was assigned a unique identifier to match their responses between the two time periods.

### Data analysis

A missing variable analysis found that less than 5% of data were missing. With the assumption that data were missing at random, a single-imputation method was used to replace missing values with a series mean. Descriptive statistics for the BASiC-QI items both pre- and post-PRIME were calculated, including means and standard deviations for each item as well as the change scores between the two time periods. Independent paired t‑tests were used to compare means pre- and post-PRIME for each item, as well as each of the three subscale totals and overall total score. A Bonferroni correction was used to adjust for multiple comparisons to account for the inflation of committing a Type I error. For the item analysis, the critical value (alpha) was divided by the number of items in the subscale (e.g., by 9 for *Beliefs and Attitudes* and *Knowledge of QI* and by 12 for *QI Skills*), whereas the critical value was divided by 4 for the t‑tests which compared subscale totals and overall total score.

Validity and reliability evidence for the instrument was assessed through an exploratory factor analysis (EFA), Cronbach’s alpha, generalizability study (G-study), and correlating scores from BASiC-QI with the QIKAT-R. Due to the small sample size, a confirmatory factor analysis was not conducted.

The Kaiser-Meyer-Oklin (KMO) test and the Bartlett’s Test of Sphericity were used to test the suitability of the scale for sampling adequacy and significance of correlation between variables, respectively. The EFA was conducted in R Studio Version 3.5.2 using the maximum likelihood estimation method using promax oblique rotation, as the correlation matrix suggested that the factors were correlated to one another. EFA examines correlations between items and the relationships among variables, exploring dimensionality of an instrument. An EFA was conducted using the pre-PRIME data, post-PRIME data, and the combined data to explore factor loadings and any differences between datasets used by collapsing items and by subscale. The Kaiser criterion was used to extract factors with Eigenvalues greater than 1, and a parallel analysis was used to confirm the number of factors.

Cronbach’s alpha was calculated as a measure of internal consistency, and was similarly calculated for both pre, post, and combined data as well as using all items and by subscale. Pearson’s correlation coefficients were calculated to examine the relationship between the three subscales.

A G-study was conducted to test the reliability of the scale in the student population to examine how the reliability might be impacted by manipulating items and subscales. G_String Version IV (http://fhsperd.mcmaster.ca/g_string/index.html) was used to calculate reliability coefficients and variance components using pre-PRIME data, post-PRIME data, and the combined datasets. The object of measurement, or facet of differentiation, was the students participating in PRIME. The facets of generalization were the subscales [3] and the items nested within each subscale (9, 9, and 12, respectively). Both items and subscales were fixed in the analyses. Absolute error coefficients were used as a conservative measure of reliability instead of relative error [18]. Using the post-PRIME data as a focus of the statistical analysis, an analysis of variance was used to estimate variance components.

Decision studies (D-study) use generalizability theory to simulate various designs and examine the impact on the reliability of the scale in hypothetical scenarios [18]. Manipulation of the facets of differentiation, in this case items or subscales, allowed for examination of the reliability of the scale if it included more subscales or items. Reliability was manipulated by both modifying subscales and items, and the absolute error coefficients (φ) were again used as a conservative estimate of reliability.

Participant QIKAT-R responses were independently assessed by two trained raters with previous QI training who were blinded to the identity of the participants as well as the time of completion (pre-PRIME or post-PRIME). Raters used the standardized QIKAT-R rubric to score each response. Inter-rater reliability was assessed using intraclass correlation coefficients.

To examine convergent validity between BASiC-QI and the QIKAT-R, independent paired t‑tests were conducted in order to compare the overall scores associated with the BASiC-QI scale prior to (pre) and following (post) the educational intervention. Similarly, paired t‑tests were used to compare the overall scores associated with the QIKAT-R scale prior to (pre) and following (post) the educational intervention, as well as the scores associated with each QIKAT-R sub question (i.e., pertaining to aim, measure, and change) pre- and post-intervention. A Bonferroni correction was used to correct for multiple comparisons, dividing the critical value by four. The idea is that both measures (BASiC-QI and QIKAT-R) should show improvement across all subscales following participation in the QI training program. Following these analyses, the overall change scores associated with the BASiC-QI scale and the overall change scores from the QIKAT-R were correlated via Pearson’s method.

This research was approved by the Hamilton Integrated Research Ethics Board in Hamilton, Canada (HIREB File #0930).

## Results

Increases were seen for each of the 30 items on the BASiC-QI tool following completion of the QI program (Tab. 1). Baseline scores (pre-PRIME) were initially high for all 9 items on the *Attitudes and Beliefs* subscale but increased following completion of the program. Except for items 3, 6, and 7 on *the Attitudes and Beliefs Subscale*, all item changes measured following PRIME were statistically significant. The largest item increase following PRIME was seen in the item assessing knowledge of PDSA cycles—a methodological hallmark of QI. Increases on subscale scores were all statistically significant, however, change scores were larger for both the *Knowledge of QI *and *QI Skills *subscales. Overall, BASiC-QI scores increased 54.99 ± 25.5 following PRIME (*p* < 0.0125; 95% CI 47.4, 60.6).

**Table 1:** Descriptive statistics for BASiC-QI scale items

	Mean (SD)				
	PRE	POST	$\Updelta$ (SD)	*p*-value	95% CI
*Subscale 1: Attitudes and Beliefs*	* 50.0 (6.59)*	* 54.8 (6.71)*	* 4.77 (7.21)*	0.000***	* 2.91, 6.63*
1. I enjoy QI	4.54 (0.850)	5.63 (1.03)	1.092 (1.10)	0.000*	0.809, 1.375
2. I am interested in QI	5.64 (0.898)	5.83 (1.03)	0.190 (1.11)	0.191	−0.097, 0.477
3. I understand the role QI plays in the health system	5.34 (1.17)	6.20 (0.605)	0.862 (1.21)	0.000*	0.548, 1.175
4. QI plays an important role in strengthening systems, such as healthcare	5.87 (0.911)	6.30 (0.743)	0.435 (1.01)	0.002*	0.173, 0.697
5. I value QI training as part of my professional development	5.71 (0.884)	6.12 (0.904)	0.405 (1.04)	0.004*	0.135, 0.675
6. I want to participate in QI initiatives as a health professional	5.71 (0.884)	6.00 (0.957)	0.288 (1.11)	0.048	0.003, 0.574
7. Applications of QI theory and methodologies can help make change to a system	5.81 (0.892)	6.15 (1.04)	0.337 (1.13)	0.024	0.045, 0.628
8. Using QI in the real world will make improvements	5.80 (0.879)	6.32 (0.676)	0.520 (0.910)	0.000*	0.285, 0.755
9. I understand the rationale for QI in the real world	5.61 (1.12)	6.23 (0.795)	0.640 (1.18)	0.000*	0.336, 0.944
*Subscale 2: Knowledge of QI*	* 25.7 (11.1)*	* 49.4 (7.41)*	*23.7 (10.2)*	0.000***	*21.1, 26.4*
1. QI theory	2.64 (1.40)	5.22 (1.14)	2.58 (1.51)	0.000*	2.99, 12.7
2. How QI is different than research	3.26 (1.68)	5.48 (1.13)	2.23 (1.63)	0.000*	2.65, 10.6
3. Systems thinking	2.98 (1.56)	5.08 (1.21)	2.10 (1.53)	0.000*	2.50, 10.7
4. 6 dimensions of quality	2.43 (1.44)	5.73 (1.18)	3.30 (1.64)	0.000*	3.73, 15.6
5. Understanding processes within a system	3.00 (1.62)	5.30 (1.23)	2.30 (1.49)	0.000*	2.69, 12.0
6. The Model for Improvement	2.50 (1.38)	5.27 (1.18)	2.77 (1.29)	0.000*	3.10, 16.6
7. PDSA cycles	2.15 (1.34)	5.83 (1.04)	3.68 (1.57)	0.000*	4.08, 18.2
8. How to measure the impact of a change	3.27 (1.53)	5.70 (0.850)	2.43 (1.51)	0.000*	2.82, 12.5
9. How change links to improvement	3.48 (1.54)	5.82 (0.701)	2.33 (1.49)	0.000*	2.72, 12.1
*Subscale 3: QI Skills*	* 27.8 (10.3)*	* 53.3 (14.3)*	*25.5 (13.5)*	0.000***	*22.0, 29.0*
1. Understanding quality gaps	2.68 (1.12)	4.42 (1.20)	1.73 (1.17)	0.000**	2.04, 11.5
2. Identifying quality gaps	2.81 (1.10)	4.72 (1.26)	1.91 (1.38)	0.000**	2.27, 10.7
3. Approach quality improvement projects	2.12 (1.09)	4.30 (1.42)	2.18 (1.38)	0.000**	2.53, 12.2
4. Understand root causes of quality gaps	2.25 (0.962)	4.13 (1.36)	1.89 (1.29)	0.000**	2.22, 11.4
5. Identifying an area for improvement	3.00 (1.11)	4.70 (1.21)	1.70 (1.27)	0.000**	2.03, 10.4
6. Application of evidence and best practices to the real world	2.81 (1.17)	4.32 (1.46)	1.51 (1.57)	0.000**	1.91, 7.47
7. Writing an aim statement	2.12 (0.975)	4.57 (1.43)	2.44 (1.38)	0.000*	2.80, 13.7
8. Using tools to identify areas for improvement	2.09 (1.03)	4.45 (1.33)	2.36 (1.33)	0.000**	2.71, 13.8
9. Using the Model for Improvement	1.77 (0.939)	4.25 (1.42)	2.48 (1.39)	0.000**	2.84, 13.9
10. Using PDSA cycles to plan and test a change	1.49 (0.866)	4.67 (1.28)	3.18 (1.35)	0.000**	3.53, 18.2
11. Designing an intervention or change	2.44 (1.06)	4.47 (1.41)	2.03 (1.46)	0.000**	2.41, 10.8
12. Use a family of measures to evaluate the impact of a change	2.18 (1.18)	4.28 (1.39)	2.11 (1.55)	0.000**	2.51, 10.5
TOTAL SCORE	103.5 (24.4)	157.5 (25.1)	54.99 (25.5)	0.000***	47.4, 60.6

*statistical significance at *p* < 0.005 level; **statistical significant at *p* < 0.004 level; ***statistical significant at *p* < 0.0125 level; Bonferroni corrections used to correct for multiple comparisons

### Construct validity

Both pre- and post-PRIME scales were suitable for factor analysis. Kaiser-Meyer-Oklin tests for the pre- and post-PRIME data were 0.860 and 0.871, respectively. Bartlett’s Test of Sphericity was significant for both periods of data (*p* < 0.001). The exploratory factor analysis extracted three factors from the pre-PRIME data and three factors using the post-PRIME data with Eigenvalues greater than 1 (Tab. 2). Parallel analysis similarly suggested three factors within the scale (Fig. 1 of the Electronic Supplementary Material). In terms of factor loadings, two items (items 8 and 9) from *Knowledge of QI* did not load particularly well onto any factor. Excluding these two items, the remaining items loaded onto each of the three remaining factors, suggesting that each subscale measures a different construct. The three factors for the post-PRIME data accounted for 68.7% of total variance. Factor 1, which loaded all items from *QI Skills*, accounted for 32.3% of the total variance. Factor 2 included loadings from all items on *Attitudes and Beliefs* accounting for 22.4% of the total variance. Finally, factor 3 loaded items on *Knowledge of QI* (with the exception of items 8 and 9 as previously mentioned), accounting for 14.0% of the total variance. Overall, the scale demonstrates multidimensionality and is suggestive that there are three constructs being measured, as initially intended.

**Table 2:** Exploratory factor analysis

	Factor loadings		
	1	2	3
*Subscale 1: Attitudes and Beliefs*			
1. I enjoy QI	0.212	* 0.644*	0.101
2. I am interested in QI	0.110	* 0.795*	–
3. I understand the role QI plays in the health system	0.189	* 0.703*	–
4. QI plays an important role in strengthening systems, such as healthcare	–	* 0.842*	–
5. I value QI training as part of my professional development	−0.140	* 0.971*	–
6. I want to participate in QI initiatives as a health professional	–	* 0.744*	0.124
7. Applications of QI theory and methodologies can help make change to a system	–	* 0.931*	–
8. Using QI in the real world will make improvements	–	* 0.878*	–
9. I understand the rationale for QI in the real world	–	* 0.973*	–
*Subscale 2: Knowledge of QI*			
1. QI theory	–	0.108	* 0.798*
2. How QI is different than research	–	–	* 0.475*
3. Systems thinking	−0.124	−0.210	* 0.997*
4. 6 dimensions of quality	–	0.124	* 0.676*
5. Understanding processes within a system	–	–	* 0.812*
6. The Model for Improvement	0.148	0.148	* 0.601*
7. PDSA cycles	–	–	* 0.714*
8. How to measure the impact of a change	0.484	–	0.133
9. How change links to improvement	0.280	0.437	0.213
*Subscale 3: QI Skills*			
1. Understanding quality gaps	* 0.934*	–	–
2. Identifying quality gaps	* 0.955*	–	−0.168
3. Approach quality improvement projects	* 0.842*	–	–
4. Understand root causes of quality gaps	* 0.693*	–	0.226
5. Identifying an area for improvement	* 0.937*	–	−0.192
6. Application of evidence and best practices to the real world	* 0.743*	–	0.212
7. Writing an aim statement	* 0.945*	–	−0.165
8. Using tools to identify areas for improvement	* 0.921*	–	–
9. Using the Model for Improvement	* 0.935*	–	0.114
10. Using PDSA cycles to plan and test a change	* 0.894*	–	–
11. Designing an intervention or change	* 0.878*	–	–
12. Use a family of measures to evaluate the impact of a change	* 0.783*	–	0.140
*Proportion of variance*	* 0.323*	* 0.224*	* 0.140*
*Variance component %*	*32.3%*	*22.4%*	*14.0%*

Extraction method: Maximum likelihood estimation with promax oblique minimum rotation

### Scale internal consistency

Assessment of internal consistency using Cronbach’s alpha showed high reliability (α > 0.9) at both pre (α = 0.962) and post (α = 0.970) periods.

Pearson correlation coefficients between each of the three subscales were positive and statistically significant (*p* < 0.01). The largest correlation was seen between the *Knowledge of QI* and *QI Skills *subscales (*r* = 0.700; *p* < 0.01). Correlations were high between the *Attitudes and Beliefs* subscale and the *Knowledge of QI* subscale (*r* = 0.655; *p* < 0.01) and *QI Skills* subscale (*r* = 0.570; *p* < 0.01).

### Generalizability study

Results of the generalizability study showed acceptable reliability coefficients in the post-PRIME data. Absolute error coefficients (φ) were 0.317 (pre-PRIME) and 0.605 (post-PRIME). The largest variance component was due to the subscale, accounting for 35.0% of the variance, which may be explained by the fact that each of the three subscales was designed to measure a different construct (Tab. 3). Learners accounted for 27.4% of variability in scores.

**Table 3:** Generalizability ANOVA table (φ = 0.605)

Source	Df	SS	MS	Variance component	% Variance
S	59	1,237.23	20.9701	0.575	27.4
D	2	885.485	442.743	0.734	35.0
I:D	27	88.5815	3.28080	0.048	2.29
S|D	118	428.948	3.63515	0.325	15.5
S|I:D	1,592	659.252	0.41384	0.414	19.8

$$G=\frac{\sigma ^{2}\left(\mathrm{s}\right)}{\sigma ^{2}(\mathrm{s})+(\sigma ^{2}(\mathrm{d})/3)+(\sigma ^{2}(\mathrm{i}\colon \mathrm{d})/90)+(\sigma ^{2}(\mathrm{s}|\mathrm{d})/3)+(\sigma ^{2}(\mathrm{s}|\mathrm{i}\colon \mathrm{d})/90)}$$= 0.605

*σ*^*2*^ variance component

*S* student, *D* subscale, *I* item, *Df* degrees of freedom, *SS* sums of squares, *MS* mean square

### Decision study

Each facet of differentiation remained fixed but was manipulated to determine the impact on reliability should the scale be modified (e.g., if the scale contained more items or subscales). A maximum reliability of 0.609 could be achieved if the scale length was doubled to include 60 items. However, a reliability of 0.595 could be achieved if the scale was reduced in half (Tab. 4).

**Table 4:** Decision study with post-PRIME data (reliability across different levels)

Subscales	Items	Total items	σ^2^ (τ)	σ^2^ (δ)	σ^2^ (∆)	Absolute error φ	Relative error Ep^2^
*Original scale*		30	0.575	0.124	0.376	0.605	0.822
3	5	15	0.575	0.139	0.392	0.595	0.805
3	20	60	0.575	0.118	0.368	0.609	0.830
1	5	5	0.575	0.408	1.152	0.333	0.585
1	10	10	0.575	0.367	1.106	0.342	0.610

*σ*^*2*^ variance component, *τ* error term, *δ* signal term, *∆* interactions and main effects

### Convergent validity

The single measures intraclass correlation coefficients for the total QIKAT-R scores between the two raters were high (T1 = 0.582, T2 = 0.731), suggesting consistency and reliability between raters. Increases in knowledge scores on the QIKAT-R were observed for all three questions following PRIME, with a mean increase on the total QIKAT-R of 7.39 ± 6.12 following PRIME (*p* < 0.05; 95% CI 5.81, 8.97) (Tab. 5).

**Table 5:** QIKAT scores

	Mean (SD)				
QIKAT-R scenario	Pre (T = 1)	Post (T = 2)	∆	95% CI	*p*-value
Aim	3.60 (2.02)	6.88 (1.98)	3.28 (2.82)	2.55, 4.00	<0.000*
Measure	4.83 (2.17)	7.23 (1.67)	2.40 (2.50)	1.75, 3.05	<0.000*
Change	3.19 (1.74)	4.93 (1.83)	1.74 (2.10)	1.20, 2.28	<0.000*
*Total QIKAT-R (/27)*	11.6 (5.01)	19.0 (4.17)	7.39 (6.12)	5.81, 8.71	<0.000*

* statistical significance at 0.0125 level; Bonferroni correction for multiple comparisons

Convergent validation using the QIKAT-R was completed with the BASiC-QI scale by calculating Pearson’s correlation coefficients between the total change scores and post-PRIME responses for items on the BASiC-QI. BASiC-QI subscale totals and overall totals were positively correlated with the scores for overall QIKAT-R scores. The largest correlations were seen between the *Attitudes and Beliefs *(*r* = 0.477, *p* < 0.01) and *Knowledge of QI *(*r* = 0.477, *p* < 0.01) subscales, however, *QI Skills *(*r* = 0.315, *p* < 0.05) and total score (*r* = 0.440, *p* < 0.01) were also positively correlated with QIKAT-R scores.

## Discussion

The results from the exploratory factor analysis suggest that the BASiC-QI scale is a multidimensional scale which measures the constructs of knowledge, skills, and attitudes towards QI. Items clustered in a meaningful way across each of the three subscales, with the exception of two items in the *Knowledge of QI *subscale. From this, we can infer that the scale is in fact measuring different latent constructs. Internal consistency of the scale was very high across all subscales and time periods. The consistently high Cronbach’s alpha scores may reflect a limitation of the sample, which is the homogeneity of the first-year medical student participants who had no prior QI training. Since several alpha scores were greater than 0.9, the scale may still have item redundancies. Inter-item correlations could be assessed to see how items correlate with one another. Items which have high correlation could be eliminated from the scale or these items could remain for future uses in measuring change between time periods. In the initial design of the instrument, items were developed in order to obtain a sample of items that represent key aspects of QI, including hallmarks of the Model for Improvement, that can be measured over time to examine changes to a trainee’s level of confidence towards QI. In this manner, content sampling is a key component of the validity argument.

Generalizability studies allow researchers to interpret the extent to which the results from a measurement taken under one situation can be generalized to another with a different level of the facet of generalization [18]. The absolute error coefficient for the post-PRIME data (φ = 0.605) suggests that BASiC-QI may be a reliable instrument to assess knowledge, skills, and attitudes towards QI. Subscales accounted for over one-third of the variance. Logically, this may be explained because each subscale is designed with items nested within each subscale to measure a different construct within the multidimensional scale. Second to subscales, students accounted for 27.4% of the variance, which is logical because students are the facet of differentiation and the object of measurement and students *should *differ in their knowledge, skills, and attitudes towards QI. Manipulation of the scale to include more items or subscales was simulated in a decision study, which found that slightly higher reliability (φ = 0.609) could be potentially achieved if the scale contained 3 subscales with 20 items per subscale. However, a 60-item scale may not be feasible to administer at repeated intervals and would also likely include a high number of redundant items. Reducing the number of items to 5 across 3 subscales would reduce the scale length by half and maintain similar reliability (φ = 0.595) to the original 30-item scale. Shortening the scale can also address potential item redundancies that may be present given that it was designed to consider the sequential steps involved in QI, and this should be further examined in research through item-total correlation matrices.

Using the existing gold standard—the QIKAT-R—correlations between the total QIKAT-R scores were correlated with BASiC-QI scores. Overall QIKAT-R scores were positively correlated with each BASiC-QI subscale total and overall BASiC-QI total scores. This suggests that BASiC-QI is measuring similar constructs to the QIKAT-R, however in a subjective manner. Observed increases in knowledge as measured through the QIKAT-R were consistent with self-reported confidence in skills and knowledge of QI. In contrast to the QIKAT-R, BASiC-QI does measure participants’ beliefs and attitudes towards quality improvement. Although some may argue that these constructs are not necessary to measure, evaluating attitudes towards QI may be predictive of later engagement in real world improvements. Fostering positive attitudes and behaviours should be considered an important aspect of any QI curriculum across all disciplines and levels of learners, as poor attitudes towards QI could result in resistance to improvement efforts in the health system.

Altogether, this research demonstrates that BASiC-QI may be useful with a population of learners early in their training where the expected levels of competence are at the level of a novice, as opposed to more advanced levels when applications of knowledge and skills are crucial. BASiC-QI may also be useful for efficient program evaluations of QI curricula or in conjunction with other QI instruments that have been previously described. Importantly, BASiC-QI does not depend on an understanding of a clinical context, which lends itself to broad use within health professions education. Ideally, BASiC-QI should be used to determine changes in knowledge, skills, and attitudes amongst learners who complete QI training. However, BASiC-QI could be used as a pre-course assessment to provide educators with a baseline understanding of their participants knowledge, skills, and attitudes towards QI and inform the curriculum and level of teaching. This instrument could also be used alongside other post-course assessment tools for preliminary feedback about the impact of QI curricula on participants, particularly for programs where faculty capacity may be a barrier to using other instruments which require trained raters.

There are several limitations in the present study. First, this study is limited by the lack of comparison or control group, as well as size and homogeneity of the sample given that participants were first-year medical students who completed PRIME with no prior formal QI training and limited clinical exposure. Given that a large majority of the class volunteered to participate in PRIME, it was difficult to recruit participants to a control arm that would complete the instruments at both time periods. Further, the factor analysis in our study was indeed underpowered, and a much larger sample would have been more desirable. Future construct validation using confirmatory factor analysis with a larger heterogeneous sample may confirm the multidimensionality of the scale and the various constructs it purports to measure. Although the generalizability of these findings is limited, use of this instrument in a larger sample, as well as in trainees at different levels (e.g., clerkship, residency) and in different disciplines, would allow for the measurement properties to be further tested and established.

Another important limitation of this research is the use of the QIKAT-R and our examination of convergent validity between these two instruments. Despite being a well-established tool that is commonly used in evaluations of QI curricula, the QIKAT-R is limited in its ability to assess QI competencies [19, 20]. Arguably, learners’ competence in QI extends beyond their ability to write an aim statement, define appropriate measures, and identify an appropriate change concept—the three components of the QIKAT-R. While BASiC-QI may be useful to assess whether or not learners have acquired basic knowledge, skills, and attitudes towards QI, it may not be as useful in evaluating more advanced QI competencies.

The use of self-reported measures in BASiC-QI aims to capture respondents’ confidence in their understanding and skills for QI as well as their perceived attitudes and beliefs about QI. However, the use of self-reported measures is a limitation of BASiC-QI, as these measures may be subjected to response bias, including social desirability bias and response shift bias. Social desirability bias refers to when participants want to ‘look good’ to others, modifying their responses based on what is perceived to be socially acceptable [21]. Response shift bias occurs when the frame of reference is influenced by an intervention, as participants’ understanding of a concept and awareness of their own knowledge or skills is shifted due to the proximity of the intervention with the assessment [22]. The main advantages of using self-reported measures include increased feasibility (i.e., ability to administer in larger samples with similar resources), reduced dependability on multiple expert raters, and the ability to collect information that may be difficult to objectively measure. As a self-assessment tool, BASiC-QI reduces the resource burden required by other established instruments that assess QI competencies. In this study, participants took less than five minutes to complete the instrument, which is a considerable reduction in the time required from participants to collect data from trainees in other instruments, such as the QIKAT-R, which took participants in our study between 8–20 min. Further, BASiC-QI does not require the time and expertise of multiple raters to score results, serving as an efficient tool to evaluate knowledge, skills, and attitudes towards QI. This tool may be used to gather estimates about baseline knowledge, skills, and attitudes towards QI, in the evaluations of QI curricula, in examining how trainee knowledge, skills, and attitudes towards QI change over time, or as an indicator for formative feedback. BASiC-QI should not be used as a summative assessment tool; however, it could be used in conjunction with other established instruments (i.e., QIKAT-R).

The Beliefs, Attitudes, Skills and Confidence in Quality Improvement (BASiC-QI) scale demonstrates that it can reliably measure self-reported knowledge, skills, and attitudes towards quality improvement in a medical student population. Importantly, BASiC-QI can be administered in programs that are not clinically situated, such as those that use education as a context for teaching QI. For health professions students who are early in their training, instruments that use clinical stems or scenarios are limited in their ability to assess QI knowledge, skills, and attitudes. BASiC-QI is not contextually bound, thus, can be utilized by training programs that apply QI to new environments. Taken together, this study indicates that this instrument may be a useful tool in the assessment of trainees throughout various stages of professional development, starting from pre-clerkship. It is recommended that the scale be used in medical learners to measure the impact of QI curricula, or to understand how trainee knowledge, skills, and attitudes towards QI change over time. Future reliability testing using other trainees, comparison groups, and medical learners at various time points is necessary to better understand the potential uses and limitations of the scale.

## Caption Electronic Supplementary Material

Supplement A: BASiC-QI Scale

Supplementary Figure 1 Parallel Analysis Plot
