# Supplementary material for: Development and psychometric evaluation of an instrument to measure knowledge, skills, and attitudes towards quality improvement in health professions education: The Beliefs, Attitudes, Skills, and Confidence in Quality Improvement (BASiC-QI) Scale
Source: Perspect Med Educ. 2019 May 16;8(3):167–76. doi: 10.1007/s40037-019-0511-8 (PMC6565662; doi:10.1007/s40037-019-0511-8)
Supplement: Supplementary file 1 — Supplement A: BASiC-QI Scale [file 40037_2019_511_MOESM1_ESM.pdf]

The Beliefs, Attitudes, Skills and Confidence in Quality Improvement Scale (BASiC-QI)

Assigned Identifier: ______________________________

Date (dd/mm/yyyy): ______________________________

1. Do you have any prior experience in quality improvement? (Circle one)

Yes No

If yes, please describe below the extent of your experience, training and QI activities:

____

____

____

____

____

____

____

____

____

____

© 2016. McMaster University. All rights reserved.

2. Please select the response which best reflects how you feel about each statement relating to Quality Improvement (QI)

	Strongly disagree	Disagree	Slightly disagree	Neither agree nor disagree	Slightly agree	Agree	Strongly agree
a) I enjoy QI	☐	☐	☐	☐	☐	☐	☐
b) I am interested in QI	☐	☐	☐	☐	☐	☐	☐
c) I understand the role QI plays in the health care system	☐	☐	☐	☐	☐	☐	☐
d) QI plays an important role in strengthening systems, such as health care	☐	☐	☐	☐	☐	☐	☐
e) I value QI training as part of my professional development	☐	☐	☐	☐	☐	☐	☐
f) I want to participate in QI initiatives as a health professional	☐	☐	☐	☐	☐	☐	☐
g) Applications of QI theory and methodologies can help make change to a system	☐	☐	☐	☐	☐	☐	☐
h) Using QI in the real world will make improvements	☐	☐	☐	☐	☐	☐	☐
i) I understand the rationale for QI in the real world	☐	☐	☐	☐	☐	☐	☐

3. I believe I am knowledgeable in the following:

	Strongly disagree	Disagree	Slightly disagree	Neither agree nor disagree	Slightly agree	Agree	Strongly agree
a) QI theory	☐	☐	☐	☐	☐	☐	☐
b) How QI is different than research	☐	☐	☐	☐	☐	☐	☐
c) Systems thinking	☐	☐	☐	☐	☐	☐	☐
d) 6 dimensions of Quality	☐	☐	☐	☐	☐	☐	☐
e) Understanding processes within a system	☐	☐	☐	☐	☐	☐	☐
f) The Model for Improvement	☐	☐	☐	☐	☐	☐	☐
g) PDSA Cycles	☐	☐	☐	☐	☐	☐	☐
h) How to measure the impact of a change	☐	☐	☐	☐	☐	☐	☐
i) How Change links to Improvement	☐	☐	☐	☐	☐	☐	☐

4. I feel *confident* in my **skills** to do the following:

	Not confident whatsoever		Moderately confident		Very confident		Extremely Confident
a) Understanding quality issues	☐	☐	☐	☐	☐	☐	☐
b) Identifying quality gaps	☐	☐	☐	☐	☐	☐	☐
c) Approach quality improvement projects	☐	☐	☐	☐	☐	☐	☐
d) Understand root causes of quality gaps	☐	☐	☐	☐	☐	☐	☐
e) Identifying an area for improvement	☐	☐	☐	☐	☐	☐	☐
f) Application of evidence and best practices to the real world	☐	☐	☐	☐	☐	☐	☐
g) Writing an aim statement	☐	☐	☐	☐	☐	☐	☐
h) Using tools to identify areas for improvement	☐	☐	☐	☐	☐	☐	☐
i) Using the Model for Improvement	☐	☐	☐	☐	☐	☐	☐
j) Using PDSA cycles to plan and test a change concept	☐	☐	☐	☐	☐	☐	☐
k) Designing an intervention or change	☐	☐	☐	☐	☐	☐	☐
l) Use a family of measures to evaluate the impact of a change	☐	☐	☐	☐	☐	☐	☐
