# Supplementary figures and images for: Development and psychometric evaluation of an instrument to measure knowledge, skills, and attitudes towards quality improvement in health professions education: The Beliefs, Attitudes, Skills, and Confidence in Quality Improvement (BASiC-QI) Scale
Source: Perspect Med Educ. 2019 May 16;8(3):167–76. doi: 10.1007/s40037-019-0511-8 (PMC6565662; doi:10.1007/s40037-019-0511-8)

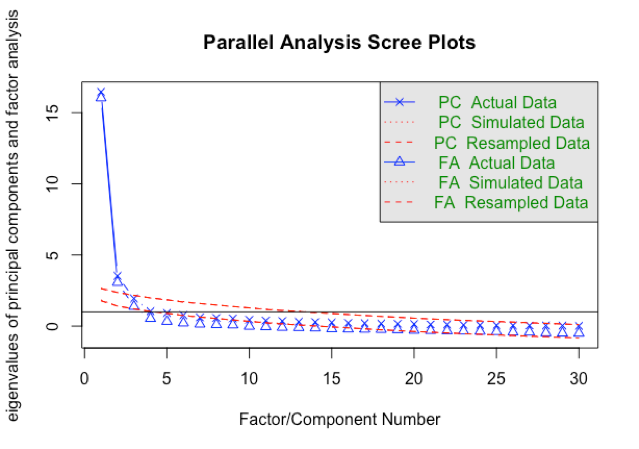

Supplement: Supplementary file 2 — Supplementary Figure 1 Parallel Analysis Plot [file 40037_2019_511_MOESM2_ESM.png]
